# The EEG Activity during Binocular Depth Perception of 2D Images

**DOI:** 10.1155/2018/5623165

**Published:** 2018-01-30

**Authors:** Marsel Fazlyyyakhmatov, Nataly Zwezdochkina, Vladimir Antipov

**Affiliations:** Kazan Federal University, 18 Kremlyovskaya Street, Kazan 420008, Russia

## Abstract

The central brain functions underlying a stereoscopic vision were a subject of numerous studies investigating the cortical activity during binocular perception of depth. However, the stereo vision is less explored as a function promoting the cognitive processes of the brain. In this work, we investigated a cortical activity during the cognitive task consisting of binocular viewing of a false image which is observed when the eyes are refocused out of the random-dot stereogram plane (3D phenomenon). The power of cortical activity before and after the onset of the false image perception was assessed using the scull EEG recording. We found that during stereo perception of the false image the power of alpha-band activity decreased in the left parietal area and bilaterally in frontal areas of the cortex, while activity in beta-1, beta-2, and delta frequency bands remained to be unchanged. We assume that this suppression of alpha rhythm is presumably associated with increased attention necessary for refocusing the eyes at the plane of the false image.

## 1. Introduction

The central brain functions underlying a stereoscopic vision were a subject of numerous studies focusing on the assessment of cortical activity during binocular perception of depth [1, 2]. The BA7 area of parietal cortex and BA19 area of occipital cortex were demonstrated to play a key role in the binocular stereo vision [3]. The perception of depth was also shown to be associated with bilateral activation of BA37 and BA39 areas of temporal cortex, and the dorsal regions of occipital-temporal cortex were found to be sensitive to the power of depth perception [3–5]. All these works focused on the process of binocular vision itself and used the stereoscope or other approaches to promote the perception of depth in the observed scenes. In our study, we explored the cortical activity during the execution of cognitive task consisted of viewing a false image in front of the plane of random-dot stereogram without a stereoscope or any other aids to binocular fusion, which requires a high concentration of attention. With this aim, we assessed the power of cortical rhythms using the scull EEG recording before and after the onset of the false image perception.

The 3D phenomenon [6] is a new and insufficiently investigated process of visual perception. For the first time, the information about novel abilities of vision was published in the auxiliary materials of our invention (patent number 226499 RU).

Our goal in the present study was the EEG activity measurement during viewing of plane images that create the depth perception and volume. To detect the depth and volume on the images, a binocular eye tracker was used. Eye tracker provides registration of eye movement. The article presents the results of measuring the alpha rhythm of nine volunteers. In the second part of this article the results of EEG activity of one subject are presented. Alpha, high-frequency beta, low-frequency beta, theta, and delta rhythms were analyzed. We found that during stereo perception of the false image the power of alpha-band activity decreased in the left parietal area and bilaterally in frontal areas of the cortex, while activity in beta-1, beta-2, and delta frequency bands remained to be unchanged.

## 2. Methods

Stereoscopic vision of a human (or stereopsis) is two eyes and two points of view. Stereopsis forms on the retinal photoreceptors two slightly displaced images (or horizontal disparity) of objects located at different distances from the eyes. The fusion of information in the visual centers of the brain from two mesh images creates a sense of the volume and the spatial location of objects in the visual field. When a planar image enters in the visual field, all images on it are located at the same distance from the observation points. Consequently, there is no disparity as a necessary condition for structuring the stereoscopic spatial perception of the images.

The third author of this work developed the educational technology of developing the ability to perceive the depth, volume, and spatial perspective on planar images, that is, 3D phenomenon, in other words, the transfer of three-dimensional perception from the objects of the habitat to the planar images, which are the results of human thinking. It is assumed that the 3D phenomenon develops as a result of training to observe the stereoscopic depth on the stereoscopic projections, stereograms, and 3D raster images. It is possible to assume that the ability to perceive the depth and volume on the planar images refers to the evolutional mechanisms of the visual system in the modern conditions of the technogenic habitat.

The binocular eye tracker with the function of determining the X-coordinates of the right and left eye is used to study the 3D phenomenon. In the first experiments on the study of the 3D phenomenon, eye movements were recorded using the SMI HiSpeed eye tracker in the binocular mode (recording frequency 500 Hz). The images were displayed on a 19′′ CRT monitor ViewSonic 90Gf, located at a distance* h* = 58 cm from the observer's eyes (resolution 1280 × 1024 pixels, 38 pixels/cm). Exposure time was from 15 to 150 sec. The software allows determining the X-coordinate of the direction of the right (*X*^R^) and left (*X*^L^) eyes in the numerical scale of the monitor. The difference Δ*X* = *X*^L^ − *X*^R^, which determines the position of the plane of the perceived image, was calculated from the coordinates. Perceived image plane is the plane on which the direction of the right and left eye concentrates on the point. If Δ*X* = 0, then it coincides with the plane of the image location, that is, with the monitor plane. Under the condition Δ*X* ≠ 0, it is either closer to or further from the monitor screen.

The binocular eye tracker allows obtaining the perception of the depth and volume of a planar image. In the conditions of three-dimensional perception of the image, perceived image plane is located behind it at a distance of 8–58 cm [6].

Formation of the 3D phenomenon elements is shown on the all planar images demonstrated to the participants in the experiment.

We believe that the 3D phenomenon correlates with the neurophysiological mechanisms of creative activity. The right hemisphere dominates with the synchronization of the biopotentials of alpha-activity, especially in the frontal cortical areas [7, 8]. On the other hand, the opposite activation effects of EEG activity of creative thinking were shown in [9, 10].

Some researchers have shown that when solving creative problems, the activity of the brain depends on the background state associated with the level of creativity [11]. Also, recent studies show that the perception of three-dimensional attributes depends on the background state of the subject. It is possible that the study of EEG activity under conditions of the three-dimensional perception of planar images will reveal correlates of divergent thinking, as was done in the study of EEG activity with simultaneous analysis of the level of intelligence and divergent thinking [7]. Data on the positive relationship between the effectiveness of divergent thinking and the level of intelligence previously were obtained [12, 13]. The great importance of right hemisphere activity when changing the effectiveness of divergent thinking was shown [8].

The above literature sources show that the functional activity of intelligence and divergent thinking in a wide range are represented in alpha, low-frequency delta, and high-frequency beta ranges of EEG activity. In addition to the experimental models of creativity are the individual characteristics of the subjects.

Although human perceptual responses to binocular disparity have been studied extensively [14], there have been relatively few studies of how human cortical activity is related to stereo depth perception. In these few studies [15–17], the authors relied on measurements of visually evoked potentials, a method that has a limited spatial resolution. Methods of EEG activity in the study of creativity was shown in [18]. Here, we explore the 3D phenomenon using EEG recording of cortical activity.

Nine female voluntaries of 20 years old participated in the study. They all have previously passed a semester cycle of training the spatial perception skills at the three-dimensional images. The scull EEG was recorded using 8-channel digital EEG system Neuron-Spectrum-1 (Neurosoft, Russia). The EEG electrode placement was organized by standard 10–20 system with a reference electrode placed unilaterally at the ear. The bipolar longitudinal row montage included eight recording electrodes: Fp1, Fp2, C3, C4, T3, T4, O1, and O2. After visual inspection of the EEG recordings using Neuron-Spectrum.NET software (Neurosoft, Russia) the data were processed and analyzed using custom-written routines in MATLAB environment (MathWorks, USA). The independent component analysis was used for removing the artifacts from the EEG recordings. Statistical analysis was performed using the MATLAB Statistics toolbox. The two-side Wilcoxon rank sum test was performed to assess the significance of differences between groups of data with the level of significance kept at *p* < 0.05.

## 3. Results and Discussion

**Figure 1:**
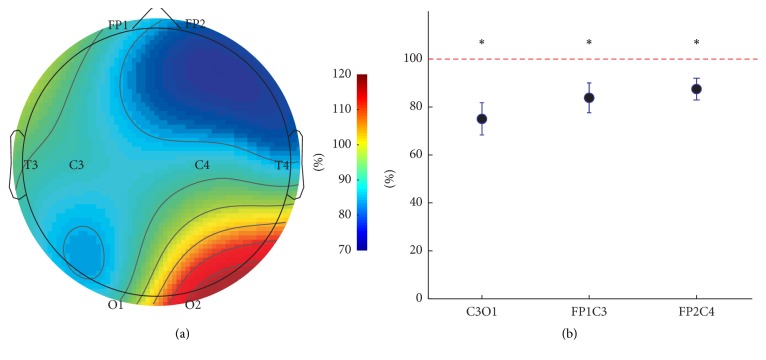
The dynamic of EEG activity in alpha frequency band during perception of depth with false objects appearing to be in front of the flat plane of random-dot stereogram without using a stereoscope: (a) the topo map of alpha-activity represented as a ratio of alpha oscillation power after/before the onset of the false image perception built for individual EEG recording; (b) averaged alpha oscillation power at C3O1, FP1C3, and FP2C4 recording sites, mean ± SE; *∗* corresponds to *p* < 0.05.

The EEG activity was recorded under three conditions: (1) with closed eyes, (2) during focusing at the plane of stereogram, and (3) during perception of the false image in front of the stereogram plane. When the eyes were closed, the alpha rhythm was recorded in both right and left occipital areas (Т3О1, С3О1, Т4О2, and С4 О2 channels) with average frequency of 9.9 ± 1.6 Hz (*n* = 9). After opening of the eyes and focusing at the stereogram plane the alpha rhythm was depressed bilaterally to the same extent. The test was signalized about the onset of the false image perception by raising up the pencil, and a suppression of activity in alpha-band was recorded after the onset of stereo perception was revealed in the frontal areas (Fp1C3, Fp2C4) and in the left parietal area (C3O1) of the cortex. Thus, the average power of alpha rhythm before and after the onset of false image perception was, respectively, 0.867 ± 0.143 *μ*V2/Hz and 0.599 ± 0.088 *μ*V2/Hz at С3О1 channel, 0.567 ± 0.121 *μ*V2/Hz and 0.457 ± 0.096 *μ*V2/Hz at Fp1C3, and 0.613 ± 0.114 *μ*V2/Hz and 0.515 ± 0.089 *μ*V2/Hz at Fp2C4 channel (*n* = 9; *p* < 0.05; Figure 1(b)).

There were not any significant changes in alpha-band activity power at other recording channels, and no changes of EEG activity in beta-1, beta-2, and delta frequency bands were detected. The changes in alpha-band cortical activity are considered to be promoted by nonspecific factors like attention, arousal state, emotions, and so on [19–21]. Thereby, we assume that suppression of alpha rhythm during the false image perception is caused by desynchronization of cortical activity associated with an increased attention, which is necessary for the viewer to refocus and fix the eyes at the plane of the false image.

**Figure 2:**
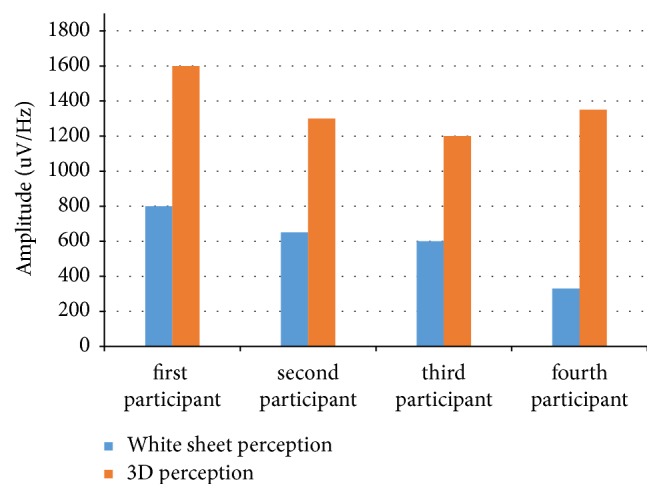
The total coherence amplitude over all leads and components of the EEG rhythms.

**Figure 3:**
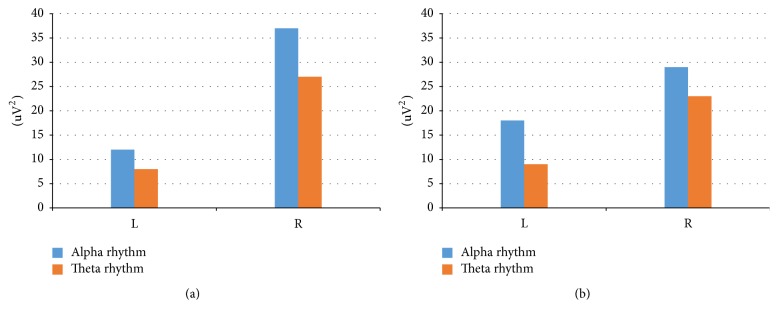
EEG power on left (L) and right (R) hemisphere; blue bar: theta rhythm; brown bar: alpha rhythm: (а) 2D perception and (b) 3D perception.

In addition to the results presented above, the following data were obtained. (1) The power spectra of the EEG activity were recorded. During perception of the 3D phenomenon in comparison with the planar perception in the entire range of EEG rhythms (eighth leads), a decreasing of power was observed. The change in power depends on the color palette of the planar image. (2) Extraction of the biorhythm index for beta, alpha, theta, and delta band components does not have a unique influence on them of various types of planar images. (3) Processing of the spectrograms of rhythms shows that, under conditions of three-dimensional perception of planar images, the total coherence amplitude over all leads and components of EEG rhythms is 1.8 and more times higher in comparison with the perception of the white paper sheet (Figure 2). (4) In the process of planar and three-dimensional perception of planar images creating effects of perception of depth and volume, the following is shown: firstly, the excess of theta rhythms power over alpha rhythms, secondly, that the theta and alpha rhythms power are exceeded in the right hemisphere in comparison with the left one, and thirdly, the detection of the increase in power in conditions of three-dimensional perception in comparison with the planar (Figure 3).

We also presented the EEG activity of the participant, who passed the first study on the binocular eye tracker [6]. When the first plane image (FPI) was perceived, the power of the spectrum of the right hemisphere in the frontal compartment was greater than for the left one 1.5 times in all leads, with a range of changes from 1.3 to 2 times. Under conditions of three-dimensional perception (i.e., the 3D phenomenon), the ratio of the spectrum power of the leads in the right hemisphere actually aligned with the left one. The average value of the ratio is 1.1; the interval is from 0.9 to 1.2. The total average ratio for all leads of the right and left hemispheres under conditions of three-dimensional perception to two-dimensional perception is 0.8 with a change interval from 0.4 to 1.0. In all leads with a planar perception of the second planar image (SPI), values for the right hemisphere are greater than for the left. The average value of the ratio is 1.3; the interval is from 1.1 to 1.4. Under conditions of three-dimensional perception, the average value of the analogous ratio is 1.1 with the variation interval from 0.75 to 1.2. For all leads, the power of the spectrum under conditions of three-dimensional perception is less than for a planar. The average value of the ratio is 0.5; the interval is from 0.4 to 0.7.

**Figure 4:**
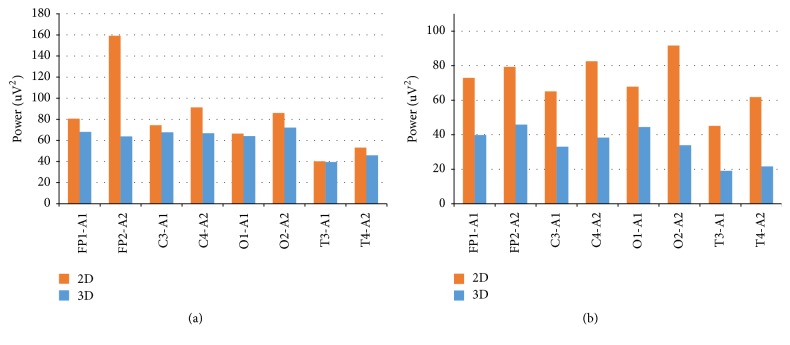
Full spectral power (all diapason): (a) first visual stimulus and (b) second visual stimulus.

Thus, under conditions of a three-dimensional perception of the presented planar images, a decrease in power spectra in comparison with planar perception and less pronounced interhemispheric asymmetry is observed (Figure 4).

Horizontally, we have frontal (FP), central (C), occipital (O), and temporal (T) leads. Odd numbers refer to the left, and even numbers to the right hemisphere. Vertically, we have power values. Brown bar represents two-dimensional (2D) and blue bar represents three-dimensional (3D) perception.

**Figure 5:**
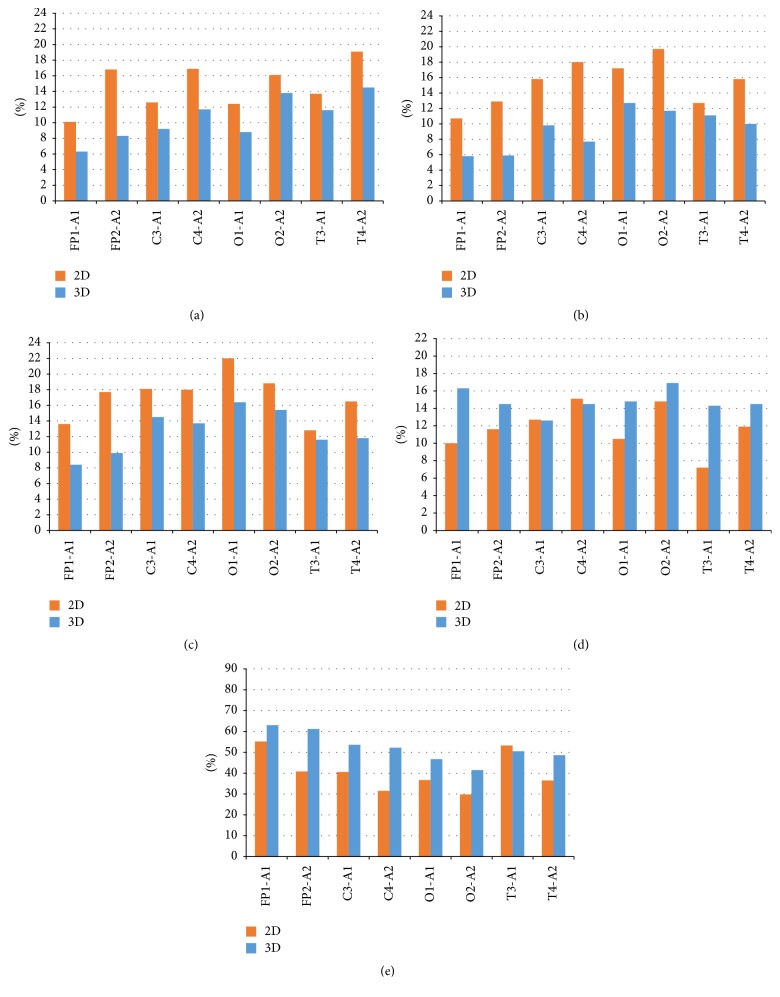
The EEG rhythm index: (a) high-frequency beta (HF); (b) low-frequency beta (LF); (c) alpha; (d) theta; (e) delta rhythms.

Figure 5 shows the graphs of the EEG rhythm index: high-frequency beta (HF) and low-frequency beta (LF), alpha, theta, and delta rhythms.

The index of high-frequency beta rhythm for both images in planar and three-dimensional perception is higher in all leads in the right hemisphere. The range of the index with a plane perception in comparison with the three-dimensional is from 10 to 21%, with the exception of the frontal lead, which is lower, about 9%. With three-dimensional perception, the index is in the range of 6–16%. It is known that high-frequency beta rhythm has a special functional significance for the integration of various signs of complex stimuli, both figurative and verbal nature. It can be assumed that this integration is reflected in the intensification of interaction between the anterior and posterior parts of the left hemisphere, which is especially pronounced in the presence of both intellectual and creative abilities.

The index of low-frequency beta rhythm with FPI perception is in the range from 5 to 16%. There is an asymmetry of the index value on the left in the frontal and occipital leads. In terms of three-dimensional perception, rhythm indices are in the range from 13 to 16%. Left-sided asymmetry is observed in the occipital-temporal leads. With a planar perception of SPI in all leads, the indices are in the range of 10 to 20%. In all leads, the value of this index in the right hemisphere is higher than in the left hemisphere. The indices of this rhythm under conditions of three-dimensional perception are significantly lower (5–12% range).

The alpha rhythm index with a planar perception of the FPI image is generally lower than in the case of three-dimensional perception. In the temporal and central leads, the right and left hemispheres have a rhythm index of one level, about 12%. In the occipital lead, values for the right hemisphere are twice as high as for the left hemisphere and in the frontal leads above the value on the left. The change interval is within 5–12%. The transition to a three-dimensional perception increases the rhythm index. It is in the range from 13 to 22%. In the temporal leads, the right and left hemispheres have approximately the same indices in the range 15-16%. It can be noted that, under the conditions of three-dimensional perception, the alpha rhythm is almost twice as high as for planar perception of FPI image in all leads with left-hemispheric asymmetry. Gradual increase in the power of the alpha rhythm with open eyes is a sign of the inhibitory condition of the oculomotor system. On the other hand, the increase in the power of the alpha rhythm may indicate a decrease in visual attention caused by the monotony of the FPI image.

With a plane perception of the SPI image, the value of the alpha rhythm index is higher than for the three-dimensional one. In the frontal and temporal leads, the alpha rhythm index is higher on the right; in the occipital lead the alpha rhythm index is higher on the left. The value of the index varies between 13 and 22%. With three-dimensional perception, the alpha rhythm index is 8–16% without a clearly expressed asymmetry.

That is, when looking at FPI image, the index of the alpha rhythm with three-dimensional perception increases; with the SPI image, it decreases almost half.

In low-frequency bands, the dynamics of the rhythm index in the case of a planar and three-dimensional perception of the images are ambiguous. However, in general, the theta rhythm index is higher for a three-dimensional than for a two-dimensional perception, especially in the right hemisphere. With a two-dimensional perception, the theta rhythm index variates in the range of 4–14%, while the three-dimensional index is 9–19%.

The change in the delta rhythm index is in the range of 40–80%. When looking at FPI image in a plane perception, the delta rhythm index is higher than in the case of three-dimensional perception (40–76%), for the SPI image is lower (38–57%). In the right hemisphere, the value of the index is clearly reduced in case of plane perception. The severity of asymmetry in three-dimensional perception is insignificant in both cases.

## 4. Conclusions

We show that during stereo perception of false image appearing in front of the stereogram plane the power of alpha-band activity decreases in the left parietal area and frontal areas of the cortex, and this suppression of alpha rhythm is presumably associated with increased attention necessary for refocusing the eyes at the plane of the false image.

The use of coherent analysis of EEG activity allows obtaining an objective evaluation of the ability of three-dimensional perception of planar images, to expand the psychophysiological features of the ability to perceive plane images with depth and volume effects.

In the future studies, it would be interesting to determine the reason underlying bilateral asymmetry in the suppression of alpha-band activity power in parietal cortical areas observed during stereo perception.

In the future, we plan to conduct experiments on recording EEG activity in the 3D phenomenon and compare them with known methods of analyzing intelligence and divergent thinking. For this, it is necessary to jointly measure EEG factors and eye movements.
